# How Social Capital is Related to Migration Between Communities?

**DOI:** 10.1007/s10680-022-09642-3

**Published:** 2022-09-15

**Authors:** László Lőrincz, Brigitta Németh

**Affiliations:** 1 NeTI Lab, Corvinus Institute for Advanced Studies, Corvinus University of Budapest, Budapest, Hungary; 2 Centre for Economic and Regional Studies, Institute for Economics, Budapest, Hungary

**Keywords:** Internal migration, Social capital, Network effects, Online social networks

## Abstract

In addition to economic and infrastructural factors, social connections of people also influence migration patterns. This influence can be attributed to the resources that are made available by social contacts: social capital, which can also be utilized in the process of migration. Based on previous literature, we identify three different aspects of social capital and test their relationship with domestic migration simultaneously. First, we analyse if the intensity of connections within communities (local social capital) restrains from migration. Second, if the intensity of connections between two communities (bridging social capital) is associated with increased migration between them. Finally, we consider, if the extent to which local community networks exhibit open or closed structures (bonding social capital) contributes to higher or lower migration rates. We create indicators for these measures using archived online social network data, covering 40% of the adult population of Hungary, and combine them with official migration data of 175 subregions. Based on point-to-point gravity and negative binomial models, we find that bridging social capital between subregions is associated with increased migration flows, but we do not find that local social capital restrains from migration.

## Introduction

Migration and its social and economic antecedents have long been the subject of scientific thinking. In the economic models, the mobility of workers would be a primary force to level up regional differences. However, in many countries, a decreasing trend of mobility was observed, which is parallel to the increasing divergence between regions (Storper, 2018). As mobility is a key prerequisite of competition in the labour market, constraints of mobility indicate a misallocation problem, leading to inefficiency of the markets and loss of potential income (Munshi & Rosenzweig, 2016). From the sociological perspective, residential mobility and immobility are a factor of structuration of societies (Coulter et al., 2013). Selective residential mobility contributes to geographic polarization and segregation (Crowder et al., 2012; Hedin et al., 2012), and decreased opportunity for mobility contributes to spatial inequalities through the intergenerational transmission of inequalities (Galster & Sharkey, 2017).

These trends are also experienced in the country of this study, Hungary. After the transition from socialism to a market economy, economic inequalities increased between regions, with unemployment in the north-eastern part of the country and the peripheral subregions (Fazekas, 2002). Differences in the level of unemployment and wages, however, had only a moderate influence on migration between regions (Cseres-Gergely, 2005), therefore the role of migration in balancing out regional differences has remained limited (Hárs, 2012).

In addition to economic considerations, social contacts are important drivers of migration (Massey, 1990; Michaelides, 2011), therefore they may contribute to maintaining this geographic divergence. Our aim, therefore, is to analyse, how social connections of communities contribute to residential mobility or immobility, and to migration between specific communities.

A difficulty in analysing the role of social connections in migration is collecting the appropriate data both on migration and on networks (Blumenstock et al., 2019; Chuang & Schechter, 2015). We overcome this difficulty by using detailed administrative data on migration and combining these with a digital trace dataset; the connections data from the Hungarian iWiW social network site’s archived database. This way we cover each officially registered migration event in the country and have a social network representation for about 40% of the adult population. We combine these data on the subregional level, enabling us to analyse migration flows between the 175 subregions.

Our approach is most related to two recent studies. Blumenstock et al. (2019) use mobile phone networks to analyse migration patterns between thirty geographic districts of Rwanda. They examine the location choice of individuals and show that having contacts at a destination area increases the likelihood of migration and that if the individual’s network connections are interconnected at these destinations, it also has a positive impact. Büchel et al. (2020) analyse residential mobility in Switzerland, also using mobile phone data. They examine both the decision on staying or moving and the location choices. They find significant network effects in both cases. They also show, that as individuals age, the relative impact of family ties increases, while the influence of friends decreases on location choice.

Because the influence of social connections on migration originates from the resources available by them (social capital) alter costs and benefits associated with migration (Massey & Espinosa, 1997); we approach these questions from a social capital perspective. Our key contribution to the literature is that we are able to analyse three different facets of social capital simultaneously. We assess the intensity of connections within communities (local social capital), the intensity of connections between communities (bridging social capital), and the extent to which local community networks exhibit open or closed structures (bonding social capital). A further differentiating element of our study is that we analyse the impact of social capital on the community level, not on the individual one. We distinguish 175 local administrative units (subregions in Hungary, corresponding to the EU LAU-1 level) with 30,450 potential connections between them.

In the following section, we present the theoretical background of the study about how social capital and migration are related and form our hypotheses. Next, we introduce the data and present the network indicators of the different social capital measures we use, followed by the description of our statistical approach. Section four presents the results, and the final section summarizes the conclusions and discusses the limitations of the study.

## Theory and Hypotheses

Social capital, according to its consensual definition across scholars is “investment in social relations with expected returns”. It includes forms of “information, influence, social credentials and reinforcement” (Lin, 1999). Concerning migration, social capital has the special value that it can be used to decrease the risks and costs associated with migration and increases its benefits too (Massey & Espinosa, 1997). Social capital can be approached from the perspective of the individuals: as a resource that is convertible to other forms of capital (Bourdieu, 1986). In a different approach, Coleman (1988) considers social capital as a property of the local network structure that consequently has a public good nature. These two facets of social capital are regarded as “relational” and “structural” (Nahapiet & Ghoshal, 1998).

Different network configurations provide different utilities for the individuals. Bridging positions between communities provide information and control benefits for the individual (Burt, 1992; Golub & Lever, 2010). On the other hand, locally dense and closed social structures have the advantage of preserving norms, reciprocity, and trust (Coleman, 1988, 1990; Putnam, 2000). These two aspects of social capital are labelled as bridging and bonding social capital (Putnam, 2000), and they presumably influence migration in different ways.

Considering migration, social contacts may provide useful information about specific localities (Coombs, 1978; DaVanzo, 1981), and learning about possible destinations increases the probability that potential migrants find an option that is attractive. In addition, new information available through social contacts may also influence one’s goals and aspirations (Coulter et al., 2013).

Further, connections may influence the costs and benefits related to migration. An important benefit^1^ comes from that social networks are important sources of job information (Granovetter, 1973), and migrants use them even more extensively than natives (Lancee, 2013). Using personal networks as a channel also results in better quality jobs for immigrants (Drever & Hoffmeister, 2008).

The benefits of social capital may also be non-monetary. Friends and relatives exchange smaller and bigger services, financial aid, emotional support, and companionship (Wellman & Wortley, 1990). In fact, Michaelides (2011) finds that these non-monetary resources have a more significant effect on individual migration decisions than monetary ones, like wage and housing cost differences. The exchange mechanism of support includes tangible dimensions, like instrumental support or companionship, and intangible ones, like emotional support (Herz, 2015). While due to modern communication technologies, emotional support is available over long distances (Ryan et al., 2008; Viry, 2012), the tangible aspect of social exchange is limited by distance (Herz, 2015; Mulder & van der Meer, 2009). Therefore, the geographic location of the contacts becomes important. Accordingly, following Kan (2007) the term *local social capital* is distinguished to refer to the households’ resources arising from social ties with people living nearby (Ermisch & Mulder, 2019). In contrast, having social network connections in distant places create *bridging social capital* between the localities.

The impact of local social capital on migration was documented by Dawkins (2006) and Kan (2007) showing that the presence of friends, relatives, and friends of children impedes the outmigration of families from the neighbourhoods. Frequent interaction with neighbours, or meeting friends and relatives also decrease the likelihood of moving out of the community (Clark & Lisowski, 2019; David et al., 2010). Having many friends nearby reduces the movements to locations further than 20 miles away (Belot & Ermisch, 2009). Zhao and Yao (2017) use the expense of wedding gifts as a proxy for local social capital in China and shows that it is negatively correlated with migration probability. Because the exchange of social support is most frequent within the close family, living close to parents was found to decrease migration propensity also in Britain (Ermisch & Mulder, 2019), and Germany (Hünteler & Mulder, 2020).

Concerning bridging social capital, the location of family members was found to be an important driver of migration, especially when they are in need of services (Michielin et al., 2008; Stokenberga, 2019). Therefore, having family members at the destination positively affects the likelihood of moving there (Hedman, 2013).

These individual mechanisms add up to the community level in different ways. First, if the local network of the community is dense because many locals have a high number of connections to each other, that restrains them from migration, and the migration rate will be lower. In addition, Glaeser et al. (2002) argue that the expected migration of an individual decreases her investment in local social capital, as when moving, these local ties are likely to be lost. However, if the migration rate in the community is high, the individuals’ expectation that others will move away also increases, which also decreases their expected return on social capital investment. In contrast, if many locals have a high number of connections at other locations, they can provide information on opportunities to them, fostering their migration. But having these networks may not only influence the behaviour and attitude of those having direct connections, but the information can spread over the local networks and may be utilized by indirect contacts as well. This corresponds to the findings of Blumenstock et al (2019) about the impact of alters’ social networks on the ego’s migration decision.

Accordingly, we predict that social capital originating from relations to local individuals is negatively associated with the outmigration rate of a community (H.1). On the other hand, we presume that bridging social capital has a positive influence on migration flows between localities (H.2).

Portes (1998) emphasizes a further function of social capital, which is social control. It is a property of tight community networks, useful to parents, teachers, and authorities to maintain compliance. For example, it allows parents to let the children play in the street as someone is always watching (Coleman, 1988) or includes norms preventing children from dropping out of school, or falling into a gang (Zhou & Bankston, 1994). In the dichotomy of bridging and bonding, these are the benefits of bonding social capital. These control mechanisms may also provide assets for the residents, but they also support the maintenance of traditional, asymmetric norms, e.g. on gender roles or family relations. These can create feelings of anxiety, frustration, and being neglected in the weaker counterparts of these hierarchies, like young women (Zontini, 2010). Altogether, we propose that the bonding social capital of communities is negatively correlated with their outmigration rates (H.3).

The effect of social capital on migration however is not a single static phenomenon, but they are rather simultaneously interrelated. When migrants settle at their new locations, they create new connections but do not break up (all) their old ties. They often visit their relatives “back home”, even after marriage and having children (Litwak, 1960; Massey & Espinosa, 1997). Communication with some of their old contacts may even increase after the migration event (Fudolig et al., 2020). Thus, they create network ties between their community of origin and destination. Consequently, every new migrant lowers the cost associated with migration to a new set of friends and relatives, creating further migration, accumulating further social capital, creating a self-reinforcing cycle (Massey & Espana, 1987; Massey & Espinosa, 1997).

## Data and Methods

Our empirical strategy is to create indicators of the different social capital measures between and within communities building on social network data and to analyse their relationship with migration.

### Data

The source of social network data is the user and network database of the iWiW online social network (OSN). iWiW was the major OSN in the pre-Facebook age in Hungary. It was founded in 2002, and gained great popularity, serving more than 3 million active users by 2009. At that time 5 million out of the 8.5 million adults used the internet in the country. Facebook arrived in Hungary in 2008 and became available in Hungarian in 2009. In 2010 the popularity of iWiW started to decrease, and it was finally replaced by Facebook, similarly to other local and global online social networks worldwide. Finally, it was shut down by the provider in 2014. All user data (registration date, gender, age, place of residence) and the links between users were archived and anonymized by the service provider in 2013 and made available for research purposes.

The source of the migration data is the Hungarian Central Statistical Office's (CSO) domestic migration data files, which include the source, the destination, and the date of each migration act in Hungary, together with information on migrants’ age, gender, and marital status.

As individuals were unidentifiable in both the social network and in the migration data, we had to aggregate these databases in order to join them. The lowest level, where it was possible was the municipalities. This is however very detailed in Hungary, providing around 3,200 municipalities. Therefore, we selected one level higher aggregation of local administrative units for our analysis, the Hungarian subregions corresponding to the LAU-1 level in the EU. One argument supporting this choice is that subregions are shown to function as local labour markets (Kertesi & Köllő, 1997), better than municipalities do (Csáfordi, 2014). Thus, they constitute the geographic unit, where people can commute to work. Further, subregions are also functional units in terms of services, health care, and education. Thus, we aggregated the individual migration events to the subregion level to combine with social capital measures calculated from the social network data. We excluded the population under 14 from the migration data, as that was the minimum age requirement for being a member of the online social network.

Next, we added data on the available services and economic characteristics of the subregions, which comes from the municipality-level statistical database (T-Star) of the CSO. It contains information in 21 different fields (demography, health care, jurisdiction, industry, trade and hospitality, business organizations, transport and communications, public administration, environmental pollution, communal infrastructure, culture and public education, housing, agriculture, unemployment, municipal aid, municipal finance, social care, tourism, taxation).

### Social Capital Measures

Several indicators were proposed to measure social capital using social network data, but there is a consensus over the simplest network indicator; the number of connections (degree) (Borgatti et al., 1998; Lakon et al., 2008). This measure was also used in studies about migration (e.g. Massey & Espinosa, 1997). We also follow this tradition, and use the number of connections, as an indicator.

However, according to our hypotheses, we differentiate *local social capital* of a community, measured as the sum of connections within a community (H.1) from the *bridging social capital*, the sum of connections between two different subregions (H.2). Note, that adding the local social capital measure of a subregion to the sum of its bridging connections gives back all the connections, its residents have.

*Bonding social capital* (H.3) refers to locally closed network structures. Local closeness on the dyadic level corresponds to relational embeddedness, that is the chance that the two parties have common partners, which was shown to increase trust between partners (Buskens & Raub, 2002; Buskens & Weesie, 2000; Rooks et al., 2000). However, the application of this phenomenon to the community level is less consensual. Norbutas and Corten (2018) measure bonding capital with network density, and Wachs et al. (2019) use the indicator of network fragmentation (modularity). For our purposes, we propose a different measure, *network clustering*. As network clustering measures the chance that two friends of a person know each other, we believe that it is more directly related to the social control over the behaviour of the ego in the community described by Portes (1998), than density or fragmentation. The clustering coefficient, however, is greatly dependent on the size of the community; it tends to be smaller in larger networks. Therefore, following Neal (2017) we standardize it by the average degree of individuals ($k$) and the size in terms of individuals ($N$) for each subregion:

1 $${\text{SC}}^{{{\text{bond}}}} = \frac{C}{{{k \mathord{\left/ {\vphantom {k N}} \right. \kern-\nulldelimiterspace} N}}},$$

where *c* refers to the (global) clustering coefficient of a subregion. As a robustness check, we also tested alternative measures of the clustering coefficient; the average local rather than the global clustering coefficient, and their non-standardized versions.

### Online Social Networks as Indicators of Social Capital

As we use online social network data to measure social capital, we have to take into consideration its particular aspects. Compared to traditional techniques of obtaining ego networks and social capital, like the name generator, position generator, or resource generator techniques (see, e.g. Van der Gaag & Webber, 2008 for an overview) the list of friends on a social network site is special in two ways. First, it sets a relatively low threshold for someone to be a member of the ego network, therefore it puts a higher weight on weak ties. Second, online social networks include latent ties, which are not active but can be converted to weak ties (Haythornthwaite, 2002). About migration, social media ties (including reactivated latent ties) provide information and assistance about the migration process and the settlement before migration, or about finding housing and employment. They also facilitate integration into the new community after the move (Dekker & Engbersen, 2014; Hiller & Franz, 2004). Thus, when measuring the relationship between social capital and migration using online social networks, we are more likely to capture the impacts associated with weak ties (like information and job referrals) than the impacts of strong ties (like help and support provided by the family).

A further consideration about OSNs is that social media offer cheap and easy communication platforms for maintaining connections, therefore they contribute to the feeling of proximity to the community of origin for migrants (Dekker & Engbersen, 2014) and generate social support for them (Chen & Choi, 2011; Ye, 2006). Even for domestic migrants, communication available on the internet contributed to maintaining their social connections (Hampton & Wellman, 2001). Therefore, having social media connections to the home community (in contrast to having offline networks only) may decrease the costs of migration. To sort out this mechanism, we include a control for the prevalence of social media use in the subregions.

### Controls

Non-network-related factors may bias our estimations if they are correlated with migration and network characteristics, therefore we must control for them in the models. The first such factor is the age profile of communities. As migration is often associated with specific events in life, such as completing studies, marriage, divorce, children leaving parents, and retirement (Chevan, 1971), age is one of the most important predictors of residential mobility (Clark & Hunter, 1992; Greenwood, 1997). Therefore, we introduce controls for the demographic profile of the subregions in the analysis. We divided the population into fourteen groups based on gender and age and added the shares of these groups as control variables.

Second, economic opportunities and available services are also important. Expected income differences may enhance migration, while amenities in neighbourhoods influence satisfaction with the residential location, and low satisfaction makes households consider relocating (Speare, 1974).^2^ These neighbourhood characteristics include density, private services like stores and restaurants; and local public goods, such as school quality, health and childcare facilities, and public safety (Kim et al., 2005; Nechyba & Strauss, 1998; Špačková et al., 2016).

To describe the infrastructure and characteristics of the source and destination subregions, we chose 25 variables about education, unemployment, housing, cultural facilities, public utilities, retail stores, economy, and health care from the T-Star database. When selecting these indicator variables, a key condition was to find a good example of a represented field (for example the number of general practitioners describes the basic public health care). We aggregated those variables to the level of subregions and then standardized them for the population.

Next, we used the double selection lasso regression to select the variables from the demographic variables and the characteristics of the subregions that are relevant controls for our model. The input variables and the coefficients of the selected controls can be found in Appendix 1.

### Analytical Model

In the analysis, we model point-to-point migration by the gravity approach. For empirical estimations, the logarithmic form of the gravity equation is used, which is:

2 $$\log M_{ijt} = \log {\text{Pop}}_{it} + \log {\text{Pop}}_{jt} + \log D_{ij} + {\text{controls}} + \varepsilon_{ij} ,$$

where ${M}_{ijt}$ is the migration between communities (*i*) and (*j*) in time (*t*), Pop is their populations, and *D* is the distance between them in terms of spatial distance or travel time.

Gravity models serve as a useful starting point for analysing point-to-point migration, as they can easily be supplemented with push and pull factors describing the source and destination areas, and have been proven to provide robust results and good model fits in the context of internal migration (Greenwood, 1997; Poot et al., 2016).

Variables corresponding to our hypotheses are also directly applicable in this framework. We can add our local social capital ${\mathrm{SC}}^{\mathrm{loc}}$ and the bonding social capital measure ${\mathrm{SC}}^{\mathrm{bond}}$ as properties of the sending subregion (push factors), while bridging social capital ${\mathrm{SC}}^{\mathrm{brid}}$ can be added for each sending-target subregion pairs. We then forward the left-hand side of the equation by one year to exclude direct endogeneity, and get the following formula:

3 $$\log M_{ij,t + 1} = \log {\text{Pop}}_{it} + \log {\text{SC}}_{it}^{{{\text{loc}}}} + \log {\text{SC}}_{it}^{{{\text{bond}}}} + \log {\text{Pop}}_{jt} + \log D_{ij} + \log {\text{SC}}_{ijt}^{{{\text{brid}}}} + {\text{controls}} + \varepsilon_{ij}$$

As controls, we include the share of iWiW users in the subregions and their urbanization. As social network connections tend to be structured by administrative regions (Lengyel et al., 2015), we add a dummy representing whether the source and destination subregions are in the same county. We include the distance (*D*) measured by travel time on road, in minutes. Further, we include the characteristics and demographic profile of the subregions that were found relevant by the double selection lasso.

When estimating Eq. (3), we have observations for each pair of subregions; however, the independent observations for the local and bonding social capital indicators refer to the subregion level. To avoid underestimating the standard errors, we, therefore, use clustered standard errors in the estimations by subregion (*i*) and (*j*).

We estimate Eq. (3) for year *t* = 2013, the last observed year in the iWiW database. We choose this year to maximize the number of observed connections. In addition, diffusion of this service was selective; it first became popular in the capital, spread to other cities later, and arrived to small villages at last (Lengyel et al., 2020). Therefore, by choosing the final year, we also minimize this selectivity.

A common problem of estimating the gravity models in logarithmic form is the occurrence of zeros. A frequent technique to evade this is to add a positive constant to the observations in order to obtain log values for the initial zeros, despite this estimator is not consistent and biased (Bellégo et al., 2021). This problem is also relevant in our case. We have 175 observed subregions that form 30,450 pairs, and only 18,450 of them have nonzero migration flows (Table 2). Therefore, we extend our analysis with count models that consider zero outcomes as well. For this purpose, we estimate a negative binomial model, which is useful in this setting, as it is not sensitive to the overdispersion problem in contrast to Poisson regression (Liu & Shen, 2014). The corresponding formula is:

4 $$E\left( {M_{ij,t + 1} } \right) = \exp \left( {\alpha + \log {\text{Pop}}_{it} + \log {\text{SC}}_{it}^{{{\text{loc}}}} + \log {\text{SC}}_{it}^{{{\text{bond}}}} + \log {\text{Pop}}_{jt} + \log D_{ij} + \log {\text{SC}}_{ijt}^{{{\text{brid}}}} + {\text{controls}} + \varepsilon_{ij} } \right).$$

One may also recognize a straightforward alternative for examining the hypotheses about the local and bonding social capital, by running the analysis on the level of the *N* = 175 subregions and contrasting outmigration volumes to these social capital measures. We regard this identification as inferior compared to the suggested ones. Because migration is negatively related to distance, at places in disadvantaged regions, surrounded by other non-attractive locations, migration could be low, and living there may also affect the social orientation of people, creating more local connections within the community. In contrast, at places where there are many attractive locations nearby, people may also form more external, and less local connections. This may create a spurious correlation between social capital and migration in a subregional level model, but not in the point-to-point approach.

### Descriptive Statistics

The regional classification provides 175 subregions which will be the frame of our analysis. The average population of the subregions is 57 thousand (Table 1). Our OSN data covers 3.3 million users, which represent 40% of the population over 14 years. The average yearly mobility by subregion is 1655 persons. Subregions have on average 3.9 million connections (thus an average user has approximately 200 connections), of which 2.2 million are within the community, and 1.7 million connect to other subregions.

**Table 1:** Descriptive statistics by subregions

	Mean	SD	N
Population (${\text{Pop}}_{i}$)	56,824	131,121	175
$\log {\text{Pop}}_{i}$	4.57	0.33	175
Migration ($M_{i}$)	1,655	3,558	175
$\log M_{i}$	3.06	0.31	175
N of OSN users (${\text{iWiW}}_{i}$)	18,909	61,606	175
Log (${\text{iWiW}}_{i}$)	4.00	0.40	175
Sum of external connections (${\text{SC}}_{i}^{{{\text{brid}}}}$)	1,685,807	4,549,794	175
$\log {\text{SC}}_{i}^{{{\text{brid}}}}$	6.00	0.36	175
Sum of local connections (${\text{SC}}_{i}^{{{\text{loc}}}}$)	2,237,650	6,645,080	175
$\log {\text{SC}}_{i}^{{{\text{loc}}}}$	6.07	0.43	175
Standardized clustering (${\text{SC}}_{i}^{{{\text{bond}}}}$)	24.21	41.01	175
$\log {\text{SC}}_{i}^{{{\text{bond}}}}$	1.25	0.28	175

log transformations are 10-based

The 175 subregions form 30,450 possible pairs. The average migration between two subregions is rather low, 9.5 persons (Table 2). The logarithmic migration flow is calculated for only those instances, where migration is nonzero, which is 18,945 out of the 30,450 subregion pairs. The average number of connections between two random subregions is 9688. We observe a positive number of connections for each subregion pair, thus its logarithms can be calculated for each case. The travel distance between two randomly selected subregions is about three hours, but actual migrants choose closer destinations, with an average driving time of 1.5 h. This is indicated by the mean distance weighted by migrations.

**Table 2:** Descriptive statistics of subregion pairs

	Mean	SD	N
Migration ($M_{ij}$)	9.52	57.84	30,450
$\log M_{ij}$	0.59	0.58	18,945
N. of connections (${\text{SC}}_{ij}^{{{\text{brid}}}}$)	9688	66,457	30,450
log ${\text{SC}}_{ij}^{{{\text{brid}}}}$	3.05	0.77	30,450
Driving distance in minutes ($D_{ij}$)	176.61	77.93	30,448
$\log D_{ij}$	2.19	0.23	30,448
Driving distance weighted by migration	85.80	61.04	18,943

log transformations are 10-based

**Fig. 1:**
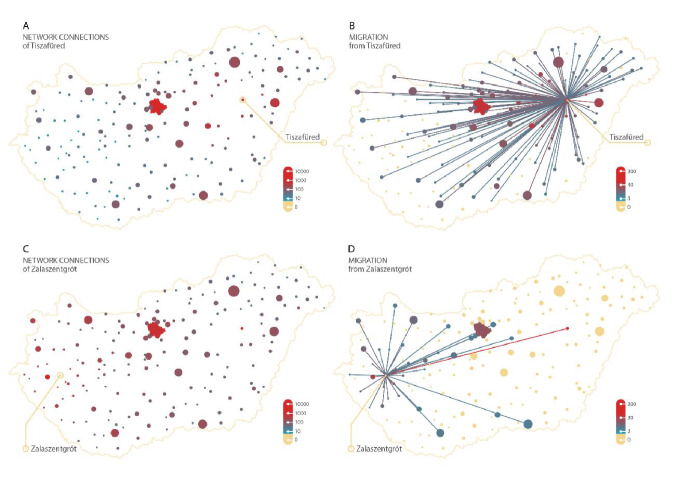
Social network connections and migration from two selected subregions. *Notes* Colours represent social network connections (panels **A**, **C**) and migration volumes (sum of 6 years; panels **B**, **D**), both on a logarithmic scale. Node sizes represent population of subregions

Figure 1 illustrates the social network connections (left panels) and migration connections (right panels) for two subregions as an example: one in eastern Hungary (upper panels), and one in western Hungary (bottom panels). Beyond the overlap between the intensity of migration and network connections, it is visible that both are related to distance, and the population of the destination subregion (represented by the sizes of the circles).

## Results

### Gravity Models

We examine the relationship between social capital and migration in four model specifications. First, we examine the measures corresponding to H1–H3 one by one (Table 3, columns 1–3), then enter them into the model simultaneously (Table 3, column 4).

**Table 3:** Gravity models on migration

Model	1	2	3	4
Log local social capital_*i*_	0.124 (0.0992)			− 0.143 (0.0984)
Log bridging social capital_*ij*_		0.667*** (0.0262)		0.666*** (0.0259)
Log bonding social capital_*i*_			− 0.225** (0.0894)	− 0.0918 (0.0875)
Log population_*i*_	0.754*** (0.0958)	0.704*** (0.0506)	0.851*** (0.112)	0.705*** (0.0623)
Log population_*j*_	0.759*** (0.148)	0.700*** (0.0906)	0.755*** (0.147)	0.698*** (0.0890)
Log *i*W*i*W_*i*_	− 0.390** (0.154)	− 0.743*** (0.0488)	− 0.205** (0.0912)	− 0.522*** (0.0155)
Log *i*W*i*W_*j*_	− 0.169 (− 0.116)	− 0.662*** (0.0692)	− 0.167 (0.115)	− 0.660*** (0.0677)
Log distance_*ij*_	− 1.050*** (0.0376)	− 0.147*** (0.0369)	− 1.051*** (0.0375)	− 0.146*** (0.0371)
*R*^2^	0.682	0.761	0.683	0.761
*N* (subregion pairs)	18,943	18,943	18,943	18,943

Coefficients, clustered standard errors in parentheses. Dependent variable: log migration_*ij*,*t*+1_

Additional controls: population rate by urbanization, share of selected demographic groups (gender × age categories), subregion characteristics, and same county dummy. Full regression table is displayed in Appendix 2

***p < 0.01, **p < 0.05, *p < 0.1

The parameter of the local social capital is not significant in any specifications, so we cannot confirm H.1, that the *local social capital* of a community, measured as the sum of connections within a community restrains outmigration.

The coefficient of the bridging social capital is consistently significant and positive, suggesting that the more intensive the network connections between subregion pairs are, the higher the migration between them is, corresponding to H.2. The coefficient suggests that a one per cent increase in social network connections is associated with a 0.66 per cent increase in migration. Adding the bridging social capital measure to the model substantively increases the R^2^ measure too, underlying the significance of its relationship with migration.

The parameter of the bonding social capital is also negative in two specifications, which is in line with H.3, however, it is only significant in those specifications, where other social network measures are not included. Therefore, our evidence supporting this hypothesis is weak.

The coefficients of our control variables correspond to expectations in such a gravity setting. The coefficients of the populations of the two subregions are positive and have approximately the same magnitude, while migration tends to decrease by distance. One can also observe that the coefficient of driving distance decreases significantly after the inclusion of the bridging social capital measure, which indicates the positive correlation between social connections and geographic proximity.

### Negative Binomial Models

As a robustness check, we estimate our models in a negative binomial form, according to Eq. (4). We specify the models similarly to the gravity models, so we first examine the measures related to H.1–H.3 one by one, and then simultaneously. We can observe that the number of observations increases from 18,943 subregion pairs to basically all 30,450 pairs in this specification, as this model also considers subregion pairs with zero migration. However, we see no difference at all in the significance and the direction of coefficients when comparing the two approaches. Substantively, the inclusion of the subregion pairs with zero migration means that in the negative binomial regression we consider the relationship between social capital and migration not only on the intensive margin (that more social capital is associated with more migration) but also on the extensive one (that more social capital increases the chance of positive migration). Finding similar effects indicates that our results concerning H.1–H.3 are robust across these approaches. Further, by comparing the *R*^2^ measures between Tables 3 and 4, we can observe that the gravity models have very high predictive power, which is lower in the case of the negative binomial ones.

**Table 4:** Negative binomial models on migration

Model	1	2	3	4
Log local social capital_*i*_	0.466 (0.319)			− 0.0527 (0.228)
Log bridging social capital_*ij*_		2.061*** (0.0473)		2.067*** (0.0473)
Log bonding social capital_*i*_			− 0.667** (0.252)	0.127 (0.175)
Log population_i_	2.724*** (0.270)	2.405*** (0.151)	2.697*** (0.278)	2.301*** (0.164)
Log population_j_	2.235*** (0.134)	2.050*** (0.122)	2.224*** (0.132)	2.050*** (0.122)
Log iWiW_*i*_	− 1.333*** (0.462)	− 2.208*** (0.156)	− 0.626** (0.273)	− 2.124*** (0.371)
Log iWiW_*j*_	− 0.245* (0.128)	− 1.784*** (0.116)	− 0.239* (0.127)	− 1.788*** (0.116)
Log distance_ij_	− 3.666*** (0.0795)	− 0.521*** (0.0908)	− 3.658*** (0.0794)	− 0.512*** (0.0908)
Pseudo-*R*^2^	0.255	0.313	0.256	0.313
*N* (subregion pairs)	30,448	30,448	30,448	30,448

Coefficients, clustered standard errors in parentheses. Dependent variable: migration_*ij*,*t*+1_

Additional controls: population rate by urbanization, share of selected demographic groups (gender × age categories), subregion characteristics, and same county dummy. Full regression table is displayed in Appendix 2

***p < 0.01, **p < 0.05, *p < 0.1

## Discussion

We examined the relationship between domestic migration and three social capital indicators created from social network data in Hungary.

Results indicate that more people move between places which are better connected with bridging social capital. From a theoretical angle, this may be the consequence that these networks provide information about potential localities, and also that friends and relatives create support and services for each other, which may influence the location choice (e.g. DaVanzo, 1981; Haug, 2008; Headman, 2013). As we measure social capital based on online social network data, which is composed of many weak and only a few strong ties, our estimated effects more likely correspond to benefits of the weak and latent ties; like information and assistance about the migration process or employment (Dekker & Engbersen, 2014; Hiller & Franz, 2004). This finding is robust across our estimations, we find it in both gravity and negative binomial models, regardless of the combination of control variables. The effect size in the gravity model suggests that 1% more connections between localities is associated with 0.66% higher migration volume.

While previous studies found that the contact with local friends and relatives decreases the mobility of individuals (David et al., 2010; Dawkins, 2006; Kan, 2007), we could not find evidence, that on the community-level high local social capital (high average degree within the community) would restrain migration. Thus, it may be the case that those individuals more likely leave a given community, who have fewer connections, but we find that this does not hold when we compare different communities.

Bonding social capital is useful for communities by generating trust (Coleman, 1988), and it increases social control too (Portes, 1998). We find only weak evidence that bonding social capital binds locals to their community by preventing migration. This may be explained by that even though control may be beneficial for some people but being under tight control in closed networks may be unpleasant for others. Zontini (2010) finds this phenomenon about Italian migrant women in the UK, but her arguments about the pressure of obligations by traditional norms may also be valid in rural Hungary to some extent. The lack of robust evidence of the effect of bonding social capital on migration may be due to its dual impact; namely providing benefits for some residents but putting burdens on others.

Further, while in our study we can document the correlation between network structure and migration, we cannot claim causality, that is migration was the consequence of social connections. Considering that the relationship between migration and social contacts is a self-reinforcing loop (Massey & Espana, 1987; Massey & Espinosa, 1997), it would be difficult to disentangle these relationships. Such exercise needs panel data, and despite our social network data having a temporal dimension (about the creation of the ties), the location of the users is recorded only in its final stage. Further, the systematic diffusion of the OSN service along the urban hierarchy questions its applicability as a proxy for change in social capital.

When interpreting the results, it is important that based on the community-level analysis, we cannot claim an individual-level relationship between social capital and migration; it only indicates that mobility is higher, where people have more bridging social capital on average. On the other hand, the fact that social capital is related to migration can be a reason, why online social network connections show a gravity property, despite the zero cost of distance in electronic communications.^3^

There are also limitations to be considered, that come from the OSN data. The iWiW social network data covers about 40% of the adult population; however, its coverage is selective by urbanization, providing lower representation in small villages. On the other hand, the availability of such a detailed, full-network level observation of social networks opens up new research opportunities about the geography of social networks and their implications, especially when combined with other methods. The recent approaches of estimating social networks from administrative records and building population-scale network data from these can be supplemented by simulations using online social networks. Such simulations could also extend ego-network-based data collections. Furthermore, the anonymity of the online social network data could be overcome by probabilistic matching approaches on the individual level.

## Lasso selection input variables and coefficients

**Table:**

Variables	Postselection coefficients
Number of corporations per capita in the source subregion	5.663
Number of corporations per capita in the destination subregion	5.8231
Number of private businesses per capita in the source subregion	− 0.0413
Number of private businesses per capita in the destination subregion	x
Corporations in industry sector (within all corporate enterprises) in the source subregion	x
Corporations in industry sector (within all corporate enterprises) in the destination subregion	x
Corporations in the agricultural sector (within all corporate enterprises) in the source subregion	x
Corporations in the agricultural sector (within all corporate enterprises) in the destination subregion	x
Corporations in service sector (within all corporate enterprises) in the source subregion	x
Corporations in service sector (within all corporate enterprises) in the destination subregion	x
Number of retail stores per capita in the source subregion	x
Number of retail stores per capita in the destination subregion	x
Number of bars and restaurants per capita in the source subregion	x
Number of bars and restaurants per capita in the destination subregion	x
Proportion of taxpayers in population in the source subregion	− 2.0375
Proportion of taxpayers in population in the destination subregion	− 1.6002
Average taxable income by taxpayer in the source subregion	x
Average taxable income by taxpayer in the destination subregion	x
Share of jobseekers over 180 days in the population in the source subregion	x
Share of jobseekers over 180 days in the population in the destination subregion	x
Share of jobseekers over 1 year in the population in the source subregion	
Share of jobseekers over 1 year in the population in the destination subregion	
The number of general practitioners per person in the source subregion	x
The number of general practitioners per person in the destination subregion	x
Number of hospital beds per person in the source subregion	1.9579
Number of hospital beds per person in the destination subregion	1.7765
Number of nursery places per capita in the source subregion	17.9497
Number of nursery places per capita in the destination subregion	x
Number of kindergarten places per capita in the source subregion	x
Number of kindergarten places per capita in the destination subregion	x
The number of primary school teachers per capita in the source subregion	x
The number of primary school teachers per capita in the destination subregion	x
Number of high school teachers per capita in the source subregion	4.8337
Number of high school teachers per capita in the destination subregion	x
Number of college students per capita in the source subregion	2.3047
Number of college students per capita in the destination subregion	3.0929
Availability of museum in the source subregion	0.1167
Availability of museum in the destination subregion	x
Share of drinking water network connected dwellings in the source subregion	0.6076
Share of drinking water network connected dwellings in the destination subregion	0.6533
Share of sewer network connected dwellings in the source subregion	x
Share of sewer network connected dwellings in the destination subregion	x
Share of gas network connected dwellings in the source subregion	x
Share of gas network connected dwellings in the destination subregion	x
Share of electricity network connected dwellings in the source subregion	− 0.08
Share of electricity network connected dwellings in the destination subregion	− 0.0813
Share of parks and green spaces in the settlements in the source subregion	x
Share of parks and green spaces in the settlements in the destination subregion	x
Number of dwellings per capita in the source subregion	x
Number of dwellings per capita in the destination subregion	x
*Demographic profile of the source subregions*	
14–19 female	x
20–29 female	x
30–39 female	x
40–49 female	x
50–59 female	x
60–69 female	x
70- female	x
14–19 male	x
20–29 male	x
30–39 male	x
40–49 male	8.7533
50–59 male	− 20.2186
60–69 male	− 2.3486
70- male	x

## Regression table displaying all control variables, gravity model

**Table:**

Log local social capital	− 0.143		0.124	
	(0.0984)		(0.0992)	
Log bridging social capital	0.666***	0.667***		
	(0.0259)	(0.0262)		
Log bonding social capital	− 0.0918			− 0.225**
	(0.0875)			(0.0894)
Log number of iWiW users in source	− 0.522***	− 0.743***	− 0.390**	− 0.205**
	(0.155)	(0.0488)	(0.154)	(0.0912)
Log number of iWiW users in destination	− 0.660***	− 0.662***	− 0.169	− 0.167
	(0.0677)	(0.0692)	(0.116)	(0.115)
Log distance between subregions	− 0.146***	− 0.147***	− 1.050***	− 1.051***
	(0.0371)	(0.0369)	(0.0376)	(0.0375)
Log population in source	0.705***	0.704***	0.754***	0.851***
	(0.0623)	(0.0506)	(0.0958)	(0.112)
Log population in destination	0.698***	0.700***	0.759***	0.755***
	(0.0890)	(0.0906)	(0.148)	(0.147)
Urbanization: share of population living in the capital	0.583***	0.578***	0.743***	0.740***
	(0.0453)	(0.0444)	(0.0939)	(0.0947)
Urbanization: share of population living in county capitals	0.0665**	0.0775***	0.167**	0.143**
	(0.0321)	(0.0294)	(0.0645)	(0.0673)
Urbanization: share of population living in towns	0.0400	0.0454*	0.0761*	0.0534
	(0.0249)	(0.0236)	(0.0435)	(0.0454)
Urbanization: share of population living in villages	0	0	0	0
	(0)	(0)	(0)	(0)
Same county dummy	0.166***	0.167***	0.552***	0.548***
	(0.0198)	(0.0198)	(0.0261)	(0.0263)
Demographic profile: 40–49 male of source	0.0773	0.165	− 0.918	− 0.269
	(1.001)	(0.987)	(1.901)	(1.935)
Demographic profile: 50–59 male of source	− 0.628	− 0.739	− 1.801	− 0.975
	(1.181)	(1.140)	(2.133)	(2.129)
Demographic profile: 60–69 male of source	0.923	0.565	1.068	0.340
	(1.014)	(1.024)	(2.013)	(2.022)
Proportion of taxpayers in population in the source subregion	− 0.637***	− 0.612***	− 0.934***	− 1.020***
	(0.169)	(0.171)	(0.296)	(0.304)
Number of corporations per capita in the source subregion	− 0.747**	− 0.578*	0.236	0.524
	(0.365)	(0.314)	(0.612)	(0.653)
Number of private businesses per capita in the source subregion	− 0.0474	− 0.111	0.135	0.152
	(0.0830)	(0.0702)	(0.184)	(0.166)
Availability of museum in the source subregion	− 0.00465	− 0.00255	0.0173	0.0119
	(0.0178)	(0.0172)	(0.0214)	(0.0248)
Number of nursery places per capita in the source subregion	3.451	3.798	7.850*	7.453
	(2.760)	(2.701)	(4.733)	(5.091)
Number of college students per capita in the source subregion	0.474*	0.524**	1.402***	1.386***
	(0.245)	(0.244)	(0.362)	(0.377)
Share of drinking water network connected dwellings in the source subregion	0.156***	0.131**	0.406***	0.442***
	(0.0590)	(0.0538)	(0.111)	(0.111)
Share of electricity network connected dwellings in the source subregion	0.00820	0.0130	0.0554**	0.0532**
	(0.0158)	(0.0153)	(0.0271)	(0.0265)
Number of hospital beds per person in the source subregion	0.109	0.355	2.746***	1.767*
	(0.657)	(0.590)	(0.959)	(1.041)
Proportion of taxpayers in population in the destination subregion	− 0.219	− 0.223	− 0.708	− 0.703
	(0.323)	(0.326)	(0.502)	(0.497)
Number of corporations per capita in the destination subregion	1.137*	1.145*	1.144	1.118
	(0.598)	(0.609)	(0.816)	(0.799)
Availability of museum in the destination subregion	− 0.0307	− 0.0308	− 0.0139	− 0.0133
	(0.0254)	(0.0256)	(0.0387)	(0.0383)
Number of nursery places per capita in the destination subregion	6.856*	6.828*	12.25*	12.27*
	(3.779)	(3.792)	(6.490)	(6.447)
Number of high school teachers per capita in the destination subregion	− 13.13***	− 13.10***	0.400	0.425
	(4.120)	(4.137)	(6.934)	(6.886)
Number of college students per capita in the destination subregion	0.893***	0.899***	1.725***	1.705***
	(0.342)	(0.344)	(0.498)	(0.493)
Share of drinking water network connected dwellings in the destination subregion	0.231	0.233	0.428**	0.422**
	(0.140)	(0.142)	(0.200)	(0.197)
Share of electricity network connected dwellings in the destination subregion	0.0313*	0.0314*	0.0808***	0.0804***
	(0.0168)	(0.0168)	(0.0287)	(0.0284)
Number of hospital beds per person in the destination subregion	− 0.155	− 0.154	2.643**	2.636**
	(0.959)	(0.963)	(1.339)	(1.333)
Constant	− 2.101***	− 2.165***	− 3.029***	− 3.210***
	(0.348)	(0.326)	(0.557)	(0.586)
Observations	18,943	18,943	18,943	18,943
*R*-squared	0.761	0.761	0.682	0.683

Coefficients, clustered standard errors in parentheses. Dependent variable: log migration_*t*+1_

****p* < 0.01, ** *p* < 0.05, * *p* < 0.1

## Regression table displaying all control variables, negative binomial model

**Table:**

Log local social capital	− 0.0527		0.466	
	(0.228)		(0.319)	
Log bridging social capital	2.067***	2.061***		
	(0.0473)	(0.0473)		
Log bonding social capital	0.127			− 0.667***
	(0.175)			(0.252)
Log number of iWiW users in source	− 2.124***	− 2.208***	− 1.333***	− 0.626**
	(0.371)	(0.156)	(0.462)	(0.273)
Log number of iWiW users in destination	− 1.788***	− 1.784***	− 0.245*	− 0.239*
	(0.116)	(0.116)	(0.128)	(0.127)
Log distance between subregions	− 0.512***	− 0.521***	− 3.666***	− 3.658***
	(0.0908)	(0.0908)	(0.0795)	(0.0794)
Log population in source	2.301***	2.405***	2.724***	2.967***
	(0.164)	(0.151)	(0.270)	(0.278)
Log population in destination	2.050***	2.050***	2.235***	2.224***
	(0.122)	(0.122)	(0.134)	(0.132)
Urbanization: share of population living in the capital	0.218*	0.208*	0.681**	0.677**
	(0.113)	(0.111)	(0.307)	(0.304)
Urbanization: share of population living in county capitals	0.0675	0.0578	0.320	0.251
	(0.0965)	(0.0898)	(0.215)	(0.224)
Urbanization: share of population living in towns	0.115*	0.0987	0.205	0.144
	(0.0638)	(0.0618)	(0.132)	(0.141)
Urbanization: share of population living in villages	−	−	−	−
Same county dummy	0.0747**	0.0756**	1.260***	1.248***
	(0.0340)	(0.0342)	(0.0470)	(0.0470)
Demographic profile: 40–49 male of source	− 2.933	− 2.152	− 4.557	− 2.912
	(2.864)	(2.953)	(5.966)	(5.883)
Demographic profile: 50–59 male of source	− 6.292**	− 5.547*	− 5.051	− 3.090
	(2.908)	(2.945)	(6.230)	(6.234)
Demographic profile: 60–69 male of source	1.652	0.402	2.900	1.513
	(2.953)	(2.850)	(6.437)	(6.296)
Proportion of taxpayers in population in the source subregion	− 1.251**	− 1.326**	− 2.798***	− 3.028***
	(0.502)	(0.518)	(0.984)	(1.006)
Number of corporations per capita in the source subregion	− 2.152**	− 1.587**	0.738	1.336
	(0.882)	(0.668)	(1.554)	(1.501)
Number of private businesses per capita in the source subregion	− 0.220	− 0.283	0.594	0.717
	(0.244)	(0.221)	(0.618)	(0.556)
Availability of museum in the source subregion	0.0264	0.0240	0.0803	0.0649
	(0.0463)	(0.0450)	(0.0588)	(0.0636)
Number of nursery places per capita in the source subregion	18.57**	18.63**	37.41**	36.00**
	(8.402)	(8.276)	(14.57)	(14.74)
Number of college students per capita in the source subregion	0.0841	0.142	3.386***	3.280***
	(0.607)	(0.642)	(1.161)	(1.164)
Share of drinking water network connected dwellings in the source subregion	0.166	0.178	1.103***	1.221***
	(0.191)	(0.177)	(0.407)	(0.404)
Share of electricity network connected dwellings in the source subregion	0.113**	0.116**	0.196**	0.182**
	(0.0459)	(0.0452)	(0.0849)	(0.0804)
Number of hospital beds per person in the source subregion	1.538	0.792	9.706***	6.908**
	(1.889)	(1.763)	(3.233)	(3.271)
Proportion of taxpayers in population in the destination subregion	0.260	0.256	− 1.704***	− 1.685***
	(0.289)	(0.290)	(0.445)	(0.443)
Number of corporations per capita in the destination subregion	1.634***	1.610***	1.683**	1.587**
	(0.341)	(0.339)	(0.684)	(0.682)
Availability of museum in the destination subregion	− 0.00932	− 0.00764	0.0614*	0.0651**
	(0.0300)	(0.0301)	(0.0315)	(0.0315)
Number of nursery places per capita in the destination subregion	23.41***	23.49***	39.69***	39.84***
	(4.265)	(4.272)	(5.655)	(5.592)
Number of high school teachers per capita in the destination subregion	− 36.34***	− 36.15***	8.551	8.327
	(4.532)	(4.552)	(7.654)	(7.599)
Number of college students per capita in the destination subregion	0.301	0.296	3.091***	3.020***
	(0.207)	(0.206)	(0.302)	(0.300)
Share of drinking water network connected dwellings in the destination subregion	0.189**	0.185*	0.963***	0.945***
	(0.0960)	(0.0958)	(0.135)	(0.133)
Share of electricity network connected dwellings in the destination subregion	0.179***	0.179***	0.268***	0.267***
	(0.0310)	(0.0310)	(0.0377)	(0.0377)
Number of hospital beds per person in the destination subregion	0.0430	0.0376	9.164***	9.090***
	(1.038)	(1.040)	(1.415)	(1.401)
Constant	− 8.460***	− 8.748***	− 11.19***	− 11.60***
	(0.583)	(0.534)	(0.995)	(0.946)
Observations	30,448	30,448	30,448	30,448

Coefficients, clustered standard errors in parentheses. Dependent variable: migration_*t*+1_

****p* < 0.01, ***p* < 0.05, **p* < 0.1

## Footnotes

1 Regarding the costs, studies of international migration consider that social networks decrease the cost of migration, and also changes the relative costs of choosing legal or illegal channels. Therefore, having social connections influence the choice of legal or illegal migration, and the use of intermediaries (Dolfin & Genicot, 2010; Orrenius & Zavodny, 2005). In the case of domestic migration however, there are no legal barriers, so, this is not a relevant mechanism.

2 However, the relationship between income levels and migration is more complex. Low-income territories are characterised by low-income occupations, which are associated with lower migration, eventually weakening the relationship between income difference and migration (Sjaastad, 1962). Selection effects may also occur. A negative selection in migration (when migration is more frequent within lower income groups) (Abramitzky et al., 2012) can be explained by the fact that the potential gains from migration are bigger for those who earn relatively less in the donating area, while a positive selection reflects the greater value placed on amenities in the target area by better-educated people (Collins and Wanamaker 2014). With respect to amenities, it must be considered that amenities raise housing prices, but high prices discourage inward migration (Glaeser et al., 2003; Potepan 1994).

3 We appreciate this comment of the anonymous reviewer of the EJP.
